# #FamilyisLove: understanding extreme life experiences and psychological profile of pre-service teachers in the Philippines

**DOI:** 10.3389/fpubh.2024.1320406

**Published:** 2024-11-18

**Authors:** January Dechavez

**Affiliations:** ^1^College of Education, Bicol University, Legazpi, Albay, Philippines; ^2^College of Education, University of the Philippines, Quezon City, Philippines

**Keywords:** mental health, psychological profile, personality variables, extreme life experiences, psychoemotional distance

## Abstract

Despite the focus of studies in other countries that emphasize the significant impact of household and school experiences on child development, in the Philippines, the few studies available tend to focus on the prevalence of suicide cases, bullying, and other school experiences that impact mental health. No explicit studies relate demographic information, relationships and past experiences, personality variables, and household treatment among Filipino students with regard to their own mental wellbeing. Thus, with the primary aim of describing the psychological profile of second-year pre-service teachers taking Bachelor of Technical Vocational Teacher Education for the academic year 2019–20 and to identify possible interventions and propose policy recommendations to promote mental healthcare, wellbeing, and resilience, the Basic Personality Inventory (BPI) results and focus group discussion interview texts were utilized for this investigation. The explanatory sequential mixed methods research was employed to undertake a study of student participants (*n* = 91) aged 18–35 years (X^¯^ = 19.9 years, SD = 2.00) using descriptive statistical tools for quantitative data derived from BPI results and textual/thematic analysis (visual and content) for qualitative data. The dominant personality variables reported as areas of weakness were thinking disorder, persecutory ideas, deviation, and self-depreciation. On the other hand, the identified areas of strength were cooperativeness, capacity for self-criticism, cheerfulness, and socially responsible attitudes. Finally, familial relationships/household treatment and previous experiences at home and in school contributed to the respondents’ strengths and weaknesses. For this reason, various mental health-related intervention activities, policies, and programs are recommended.

## Introduction

Students’ wellbeing is a key global consideration that needs attention to support both Sustainable Development Goals (SDGs) 3 and 4. SDG 3 highlights ensuring *healthy lives and promoting wellbeing for all at all ages*, while SDG 4 espouses *ensuring inclusive and equitable quality education and promoting lifelong learning opportunities for all*. In a recent progress report of the UN Secretary-General on the SDGs, there was an observed stagnation and regression due to the influence of different intervening factors arising out of the pandemic. Thus, different higher education institutions have initiated efforts to improve academic systems. Researchers all over the world also explore different aspects for further investigating the features and factors that influence student wellbeing and how it impacts academic performance, in particular, and their university life, in general. For instance, Cobo-Rendón et al. (1) emphasized how psychological wellbeing influenced academic performance. Sanci et al. (2) also revealed the many mental health concerns of students that affect their academic performance.

Demographics was another general consideration in identifying students’ mental, psychological, and emotional health. In their study conducted on school children in India, Singh et al. (3) revealed that students from rural areas and students from private schools both have higher scores on wellbeing. It also showed that as an adolescent ages, their wellbeing decreases. Thus, this study investigated one important academic stakeholder—the preservice teachers. Their psychological profile and extreme life experiences were explored to serve as a foundational reference for various interventions and policy recommendations.

In the Philippines, general wellbeing has been studied, and efforts to provide mental health services through the National Mental Health Program of the Department of Health have—been established. Reviews on mental health studies in the country reveal the evolution of designs-from case studies and literature reviews to intervention research and large-scale surveys (4–7). Various social science methods-have been utilized to create a narrative that establishes the status of the mental wellbeing of students in the country. However, no particular research emphasized how students, primarily pre-service teachers in the technical-vocational field, understand their own mental wellbeing. In this context, this mixed method study was conducted to identify how students perceive their own mental, psychological, emotional, and social health and what interventions and programs could possibly be institutionalized to support the mental wellbeing of technical-vocational pre-service teachers in the university. Primarily, this article aims to (a) describe the psychological profile of pre-service teachers in terms of (1) family demographics, (2) extreme life experiences narratives (ELEN) [Childhood and Adolescence], (3) personality variables, indicating the most dominant areas of personal strength and vulnerability as measured by the Basic Personality Inventory; (b) identify factors that may have contributed to the student’s strengths and vulnerabilities; (c) identify possible interventions suitable for their needs; and (d) propose policy recommendations to promote mental healthcare, wellbeing, and resilience.

## Literature review

Wellbeing is generally associated with the state of happiness, health, and prosperity of an individual. Thus, wellbeing in an academic environment is understood to refer to subjective feelings, living conditions, and experiences. Considering how adults view their own wellbeing, a number of studies reflected that children and adolescents have their own perspectives of their personal wellbeing (8). Moreover, the validity and reliability of the subjective (personal point of view) wellbeing of children and its high correlation to objective (external factors/relationships) wellbeing were widely accepted for years. Mental and psychological wellbeing for all ages was the focus of various research studies conducted in different contexts (9). Sweeting and Hunt (10) suggest that subjective socioeconomic status (SES) has an impact on a child’s holistic wellbeing. This is in agreement with Mateu et al. (11) who affirm that family and poverty are two things that strongly correlate to one’s happiness.

For this study, the psychological profile was the major consideration in the wellbeing of technical-vocational pre-service teachers. The psychological makeup of the pre-service teachers could affect their mental health. It is a state of wellbeing in which individuals recognize their abilities and limitations and are able to cope with the normal stresses of life (12). It enables them to work productively and fruitfully individually or with a team and make significant contributions to their communities. It implies optimal functioning in all facets of life at home, in the workplace, and in other social contexts. Certain disturbances in the daily normal functioning of the brain and stress coping mechanisms could lead to mental disorders. However, adverse mental health conditions can be prevented. Early identification, skilled assessment, and access to effective treatments at the early stage of illness offer real opportunities for recovery. Many degrees of recovery are possible for mentally disordered individuals, such as medication, psychotherapy, good coping or adaptive skills, a strong support system, and excellent health care. While the onset of mental ill-health often starts in childhood, adolescence, and young adulthood, these issues are not as endemic in basic education as in the realm of higher education, where its prevalence is reportedly on the rise at a level that is upsetting and exceeds service capacity.

The legislation of Republic Act 11036, otherwise known as the Philippine Mental Health Act of 2018, reflects the government’s commitment to providing a more holistic approach to healthcare. The creation of Hopeline 2016, a crisis support hotline accessible for those who may be in need of counseling, psychological, and or psychiatric services, is an equally commendable step at the macro level. The law mandates the provision of mental health services in schools, not just in hospitals and primary healthcare settings, and training of teachers and organic personnel to acquire knowledge of mental health to promote mental health education in schools and workplaces. These efforts will likely effect a new wave of understanding mental ill-health as an invisible illness, then spoken only in whispers. Moreover, the current initiative from the House of Representatives to propose House Bill 6,768, known as the Universities and Colleges Mental Health Act, similarly mandates the provision of valued, needed, and essential services for the promotion of mental health, including the hiring and training of additional university-based mental health personnel is a welcome legislative agenda in congress. Such a measure is very sympathetic to appeals from mental health professionals for greater accountability on the part of administrators of state universities and colleges to ensure the health and wellness of the school’s workforce and the various communities it serves. The bill also aims to adequately address the mental, emotional, and developmental needs of Filipino students to lessen, if not eliminate, various impediments to learning and enhance the latter’s preparedness and ability to learn, cope with failures, and deal with disruptive life events.

In formulating the mental healthcare and resilience program for the colleges offering teacher education programs, the major theoretical anchors for this research were Barbara Fredrickson’s Broaden and Build Theory of Positivity (74) which focuses on strengths rather than disabilities or deficiencies orientation, the use of positive psychotherapy as well as the World Health Organization’s updated standard protocols for providing multi-modal, multi-tiered levels of mental health interventions. The research adopted was guided by the organizational resilience theory, recognizing how crucial it is for an organization to stay healthy and well in all respects, one that survives over the long term and flourishes through the test of time (75). Without this flexibility, the institution may be doomed in the face of multiple challenges. As Denyer (13) further substantiates, organizational resilience is “the ability of an organization to anticipate, prepare for, respond and adapt to various incremental changes and sudden disruptions to survive and prosper in all aspects of governance.”

In terms of the psychological profile and mental wellbeing of pre- and in-service teachers in the technical-vocational field, global initiatives focused on the awareness and knowledge of instructors before and after being hired in an institution (14). Psychological factors in extreme environments (15, 16) were also studied by experts but not so much focusing on pre-service teachers specifically of technical-vocational background. Studies on the wellbeing of technical-vocational training schools were similarly conducted, focusing mostly on belongingness and comparing academic and vocational settings for secondary school students and teachers (17–20, 73). Efforts to improve services in technical-vocational training institutions due to technological advancement and innovations have been initiated in the academia.

In the review conducted by Chinedu et al. (21), they emphasized the pivotal role that teachers in technical and vocational education (TVE) play in improving societal wellbeing and community development. Philippines also recognizes the importance of technical-vocational skills by institutionalizing the National System of Technical Vocational Education and Training (NSTVET) (22, 23). Thus, before the actual teaching tasks in the workplace, pre-service teachers in the field of technical-vocational education are exposed to rigorous mental and physical training because, as Tilak (24) mentioned, vocational education advocates for a “flexible labor force” that makes workers more productive. Besides, the implementation of K–12 curriculum in the Philippines with technical-vocational track in senior high schools emphasizes the demand for holistically qualified teachers trained under the Bachelor in Technical-Vocational Teacher Education (BTVTEd) program (25, 26, 76). BTVTEd graduates, as stated in CMO 79, 2017, are teachers who are “equipped not only with a strong theoretical understanding of teaching and technology but also with practical exposure in the industry.” Therefore, it is highly relevant to understand not only the cognitive ability of pre-service teachers but, more so, their psychological profile to prepare them for the tasks ahead of them during their training and when they finally enter the actual teaching field.

With the pressing mental health issues among students not only in the Philippines but all over the world, this study can add nuance to the existing narrative on mental and holistic wellbeing primarily of pre-service teachers in the technical-vocational area. Furthermore, being a comprehensive study on wellbeing that uses both quantitative and qualitative methods and data is also an additional contribution of this particular research.

## Materials and methods

This section provides the details of the research design adopted to answer the specific questions guiding this study. It also includes the means of collecting the data, selecting the sample and site for research, and the approach for data analysis. Finally, it closes by directing the readers toward the limitations and potential problems associated with this particular investigation.

### Research strategy/study design

This particular study is interested in an in-depth investigation of the psychological profile of technical-vocational pre-service teachers that influences their mental wellbeing. In essence, the researcher is concerned with the exploration of different demographic factors, primarily familial relationships, including extreme life experiences and personality variables, that influence students’ mental health. Quite a few empirical research strategies (27–29) are available to carry out mental health research. However, considering the available time, data, measure, and scope of this study, an explanatory sequential mixed method design was used (27, 30). Upholding the interpretative and critical ontologies (31), gathering both qualitative and quantitative data provided the researcher with a better understanding of the study.

As Creswell (27) noted, the mixed method research consists of “merging, connecting, building, and embedding” (p. 537) numerical and non-numerical data. Simply put, mixed method study involves mixing of data, integrating analysis, and triangulating results to fully understand a chosen topic. Creswell and Clark (30) discussed the development and debates that incorporate mixed methods design in educational and social science investigations. In relation to understanding the psychological profile of technical-vocational pre-service teachers and the factors that influence their areas of strengths and weaknesses in the personality variable according to BPI results, the most appropriate type of mixed method research for this study is the explanatory sequential mixed method design.

Explanatory sequential design involves collecting data sequentially in two phases (27, 77). This design, considered as the most popular form of mixed methods approach in educational research, is also called a two-phase model (78). It consists of first collecting quantitative data and then gathering qualitative data to help explain or elaborate on the quantitative results. In this study, the Basic Personality Inventory Questionnaire provided numerical data, while the FGD notes with open-ended guide questions delivered non-numerical detail from the respondents. Considering the collected data, the inductive strategy was adopted to deeply explore the concepts that concern personality variables and extreme life experiences. Induction happened by collecting and analyzing data in response to the research questions.

This mixed-method research maximized two dominant design elements, namely, explanatory and triangulation (27, 29, 30). The explanatory design was reflected in administering the BPI Questionnaire at a specific college and time. Then, it was followed by the analysis, interpretation, and conclusive findings arrived at after studying the results of the questionnaire and the data gathered in the FGD. Finally, triangulation happened by using quantitative and qualitative data as well as mixed methods and analysis.

### Research instrument

This study utilized the Basic Personality Inventory (BPI) (32), which is a construct-based inventory designed to measure traditional areas of pathological content. BPI, as concluded by Holden and Fekken (33), is a psychometrically sound, relatively concise self-report instrument assessing the broad domain of psychopathology. Holden et al. (34) examined the psychometric properties of the BPI scales using 112 adult patients and found that the scales were internally consistent and possessed both convergent and discriminant validity.

“The BPI is a 12-scale, 240-item, true/false self-report measure of the general domain of psychopathology. It is a multiscale inventory that assesses psychological problems. The inventory is appropriate for adults and adolescents and for both nonclinical and clinical populations. The BPI requires a grade 5 reading level and can be completed in approximately 30–40 min. The BPI’s 20-item scales assess *hypochondriasis, depression, denial, interpersonal problems, alienation, persecutory ideas, anxiety, thinking disorder, impulse expression, social introversion, self-depreciation, and deviation* (33).”

The scale for this instrument is clustered into five categories: (i) cognitive style and infrequency—denial and deviation; (ii) personal cognitive adjustment includes persecutory ideas and thinking disorder; (iii) personal emotional adjustment—depression, anxiety, and hypochondriasis; (iv) social and self-perception includes self-depreciation and social introversion; and (v) and antisocial orientation—interpersonal problems, alienation, and impulse expression.

### Data collection (BPI test administration and FGD)

Selecting the means for collecting empirical data is as important in choosing the suitable research strategy. The researcher examined both quantitative and qualitative data. Due to time constraints and the scope of the study, the data needed were primarily obtained through the BPI questionnaire and FGD notes and textual data.

The data were collected after obtaining the ethics approval and consent from the concerned offices and the dean and program chairman of the institution of this study. Students were then given the opportunity to assent to be part of the research after parental consent was secured before administering the survey. The pen and paper questionnaire was answered in a designated room within the college separated from the non-participating students.

The survey was administered to 91 s-year BTVTED students aged 18–35 years from the participating college by the researcher accompanied by the associate dean and program adviser, within a 40- to 60-min period. To maintain anonymity and confidentiality, participants were instructed to place the survey instrument in a sealed envelope immediately after answering all the questions. Students were encouraged to answer each question individually and to avoid talking to their peers to maintain the confidentiality of the self-report. After the test administration, test profiling ensued to determine individual areas of personal strength and vulnerabilities. Students who obtained within the range of high up to the clinically significant threshold or range of scores on the different scale dimensions were organized into several focus groups, and information on family demographics, extreme life experiences narratives as well as the factors that contributed to the student’s psychological profile was obtained. The respondents’ test data vis-à-vis the narratives culled from the FGDs formed the researchers’ basis for the identified intervention modalities and proposed policy recommendations.

### Sampling/participants

To gather different data from a specific group of people considering time and geographic limitations, this study used purposive sampling to approximate a relatable sample of students in the university. Different students from different home environments, cultures, and fields of specialization but taking the same course participated in this study. The college and year level were deliberately chosen, considering that this is the first cohort of K–12 students from a technical-vocational course at the university. Furthermore, as emphasized by Cobo-Rendón et al. (1), affective wellbeing decreases or increases during the second academic year. All the enrolled second-year BTVTEd students (*n* = 91) participated in the BPI test. Students self-identified their gender and age in the questionnaire. Out of the 91 students, FGD notes and results of only 49 students (54%) were retrieved and utilized for the thematic analysis in this study.

Demographic information, specifically parental status for this sample, is also reported. Demographic information is essential in any empirical study on mental wellbeing as suggested by the existing literature (35–43).

### Data analysis

The researcher collected and examined quantitative and qualitative data using BPI (32). Microsoft Excel for Microsoft 365 version 2009 was used to calculate and report descriptive statistical data on the personality variables after manually scoring the BPI results of the respondents, the gender and age differences, and the reported familiar relationships. Descriptive (frequencies and cross-tabulations) statistics for analyzing the different variables such as age, gender, familial relationships, areas of strengths and weakness, and the personality that is associated with the psychological profile primarily mental health of technological-vocational major pre-service teachers were employed.

Thematic analysis (44) was undertaken to determine the common themes among the drawings and text provided by participants. Furthermore, NVivo (45–48) version 12 was used for data cleansing that aided the researcher in organizing the raw statements from the respondents.

Finally, statements given in Filipino language were translated by the researcher before undertaking data analysis. Both the literal (word for word) and free (sense for sense) translation approaches (49) were used in the treatment of the textual data. Data were collected from students who participated in the FGD wherein the researcher asked them to assign a symbol for each family member to illustrate the relationship within the family. They were also provided an avenue to share their drawings highlighting their extreme life experiences.

The robustness of data analysis is anchored on the thematic analysis. As Braun et al. (50) discussed, thematic analysis is a method for systematically identifying, organizing, and offering insights into patterns of meaning across a data set. The emphasis is on the identification of shared or collective meanings and experiences in the way the topic is discussed by the respondents. Finally, triangulation was performed using various forms of methods and data that supported the study, such as the survey questionnaire and test results using the BPI, statements, and drawings gathered during the FGDs, and the relevant documents used in the review of the literature.

## Results and discussion

### Psychological profile of the pre-service teachers in terms of personal and family demographics

The participants for this study were 18–35 years old (X^¯^ = 19.9 years, S.D. = 2.00). In the sample, 100% (*n* = 91) of the pre-service teachers took the BPI test while from 54% (*n* = 49) the FGD data were retrieved; both the results were used for thematic analysis. In addition, the dominant gender in this sample was women (65%, *n* = 59), and 34% (*n* = 31) self-identified as men and one respondent did not indicate his/her gender.

In terms of family background of the respondents as retrieved from the FGD data (*n* = 49), the pre-service teachers under study came from families with one child to eight children (X^¯^ = 4.1, S.D. = 1.77) wherein 69% (*n* = 34) identified their parents as married. Ten percent (*n* = 5) explicitly shared that their parents are married and at the same time living together.

Another 10% (*n* = 5) of the student-respondents have parents who are separated and the remaining 10% are widowed (*n* = 2), not stated (*n* = 2), and not married (*n* = 1) (Table 1).

**Table 1:** Personal demographics.

	*n*	%
**Age**		
18	1	1%
19	42	46%
20	38	42%
21	4	4%
22	1	1%
25	1	1%
27	1	1%
35	1	1%
Missing Data	3	3%
**Gender**		
Male	31	34%
Female	59	65%
Missing Data	1	1%
**Number of children in the family**		
1	1	2%
2	10	20%
3	8	16%
4	12	24%
5	8	16%
6	5	10%
7	2	4%
8	3	6%
**Parents marital status and relationship**		
Married (living together)	5	10%
Married	34	69%
Not married	1	2%
Separated	5	10%
Widowed	2	4%
Not stated	2	4%

### Psychological profile of the pre-service teachers in terms of extreme life experiences narratives

#### Childhood

**Figure 1:**
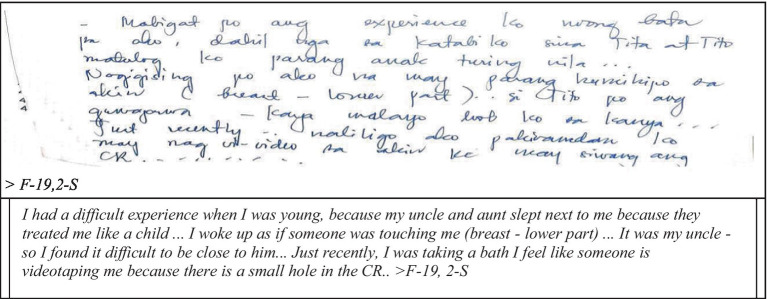
Sexual violence.

The respondents of this study were able to recall good memories of their childhood except for a few students who had extreme negative and traumatic experiences such as groping. A pre-service teacher in the study explicitly shared her traumatic childhood experience involving her very own uncle (Figure 1).

Sexual assault or sexual violence such as groping is an intentional sexual act of an individual without the consent of the person being touched (51, 52). Previous studies established the association of sexual violence to the mental health of the victim (12, 53, 72, 79). Furthermore, the depressing and self-deprecating thoughts could haunt the sexually assaulted individual in their lifetime. As experienced by the respondent, the fear of being an object of voyeurism influences her behavior at home as well. The feeling of not being safe with one’s own relatives also has an impact on how the individual treats other people. In this particular narrative, it is therefore imperative for an educational institution such as Bicol University to intentionally listen and, if possible, act on the extreme life experiences of students, which in one way or another influence not only their academic performance but more so their psychosocial behaviors.

**Figure 2:**
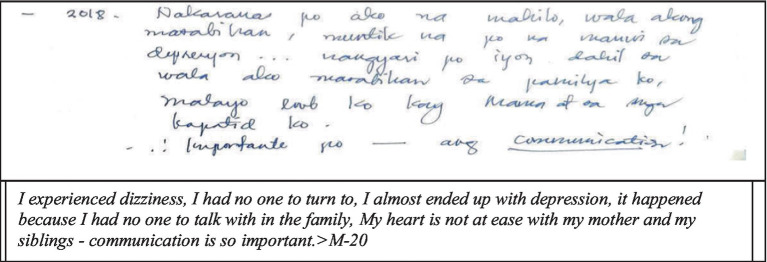
Depression.

Another extreme life experience narrative disclosed by the respondents of this study during their adolescent years is depression. Massive public attention has been given to depression as a pressing mental health issue not only among adolescents but even among adults both in developing and developed countries (54–59) (Figure 2).

**Figure 3:**
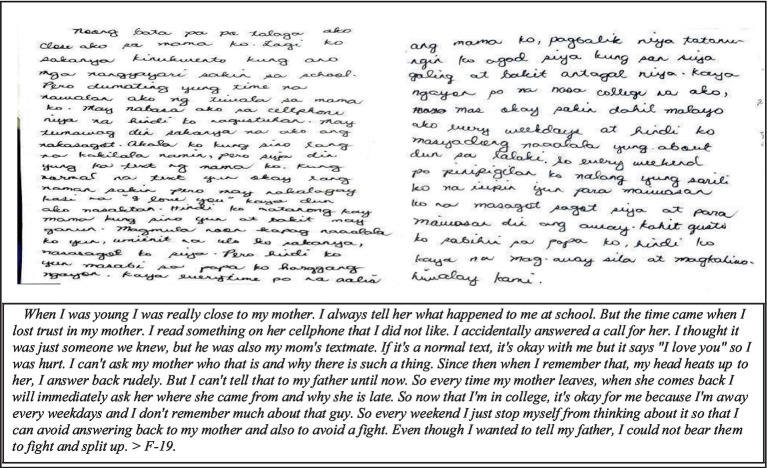
Unfaithfulness/dishonesty of parent(s).

Another dominant narrative considered as extreme by one of the respondents is unfaithfulness of a parent. Parental conflicts, as shared by Cooklin (60), witnessed by children influence how an individual behaves outside the house. In the case of the respondent in this study, she is torn between telling the truth to her father or hiding it to keep the family together. The single incident of discovering the disloyalty of her mother put a barrier to her relationship not only with her mother but also with her father (Figure 3).

### Psychological profile of the pre-service teachers in terms of personality variables, indicating the most dominant areas of personal strength and vulnerability as measured by the basic personality inventory

Table 2 discloses the personality variables of the respondents based on the BPI results. Eighty-two percent suffer from thinking disorder, while 81% of the respondents are subjected to persecutory ideas. Further studies related to suspiciousness/persecutory ideas and thinking disorder/delusional ideas among pre-service technical-vocational teachers should be conducted to clearly identify the factors that influence its development. In Masillo et al. (61) study, bullying is one of the dominant factors that leads to persecutory ideas and thinking disorder of the victims. On a positive note, 77% do not experience interpersonal problems. As future teachers, this is a good personality indicator as they know how to cooperate and respect authorities. Their regular laboratory group activities may have influenced the way they relate to each other, thus, developing collaboration and overcoming interpersonal problems.

**Table 2:** BPI result.

Variables	TS 0–59		TS 60-above	
	*n*	%	*n*	%
Hypochondriasis	40	44%	51	56%
Depression	64	70%	27	30%
Denial	67	74%	24	26%
Interpersonal problems	70	77%	21	23%
Alienation	58	64%	33	36%
Persecutory ideas	17	19%	74	81%
Anxiety	40	44%	51	56%
Thinking disorder	16	18%	75	82%
Impulse expression	38	42%	53	58%
Social introversion	47	52%	44	48%
Self-depreciation	34	37%	57	63%
Deviation	24	26%	67	74%

Number of variables with T-Score 70 and above	*n*	%
9	2	2%
8	3	3%
7	5	5%
6	12	13%
5	9	10%
4	5	5%
3	11	12%
2	15	16%
1	19	21%
0	10	11%

The top three most dominant areas of personal strength as measured by BPI are cooperativeness, capacity for self-criticism, and cheerfulness. Table 3 shows that 77% (*n* = 70) of the respondents are cooperative, obedient, and respect authority. Seventy-four percent (*n* = 67) are emotionally responsive. These pre-service teachers are open to a discussion of unpleasant topics and avoid impressing other people. At the same time, 70% (*n* = 64) of the participants of this study are confident, cheerful, and persistent despite the challenges or issues around them. Furthermore, they similarly exhibit a positive attitude about their future.

**Table 3:** Strength.

Variables	*n*	%
Cooperativeness	70	77%
Capacity for self-criticism	67	74%
Cheerfulness	64	70%
Socially responsible attitudes	58	64%
Social extroversion	47	52%
Composure	40	44%
Good physical health	40	44%
Self-control	38	42%
Self-confidence	34	37%
Commonality	24	26%
Trustfulness	17	19%
Reality of thinking	16	18%

Conversely, as shown in Table 4, 82% (*n* = 75) of the respondents are markedly confused, easily distracted, and disorganized. They shared that they cannot even remember simple things in their day-to-day life. They cannot concentrate and maintain sensible conversations because they found it difficult to distinguish daydream from reality. Concurrently, 81% (*n* = 74) believe that certain people are against him and are trying to make life difficult and unpleasant. This group of respondents do not trust others and feel threatened. Another personality variable that indicates the most dominant areas of personal vulnerability is deviation. Seventy-four percent (*n* = 67) display behavioral patterns very different from their peers and self-reported unusual behaviors and pathological characteristics.

**Table 4:** Vulnerability.

Variables	*n*	%
Thinking disorder	75	82%
Persecutory ideas	74	81%
Deviation	67	74%
Self-depreciation	57	63%
Impulse expression	53	58%
Hypochondriasis	51	56%
Anxiety	51	56%
Social introversion	44	48%
Alienation	33	36%
Depression	27	30%
Denial	24	26%
Interpersonal problems	21	23%

The specific predictors for the personality variables both as areas of strengths and vulnerabilities should be taken into consideration in future studies. Non-clinical factors such as familial relationships, which are the focus of this present research, could be explored statistically for a more generalizable result beneficial to pre-service teachers in the technical-vocational area all over the country.

### Factors that may have contributed to the students’ strengths and vulnerabilities

#### Strengths

The factors that may have contributed to the students’ strengths are familial bonds or close family ties, open communication, and faith or belief in God or a Greater Being. On the other hand, the respondents’ vulnerabilities as reflected from the gathered FGD data may have been influenced by their upbringing such as coming from a broken family, trust issues because of previous experiences, and the feeling of psychoemotional distance from other family members and friends.

**Figure 4:**
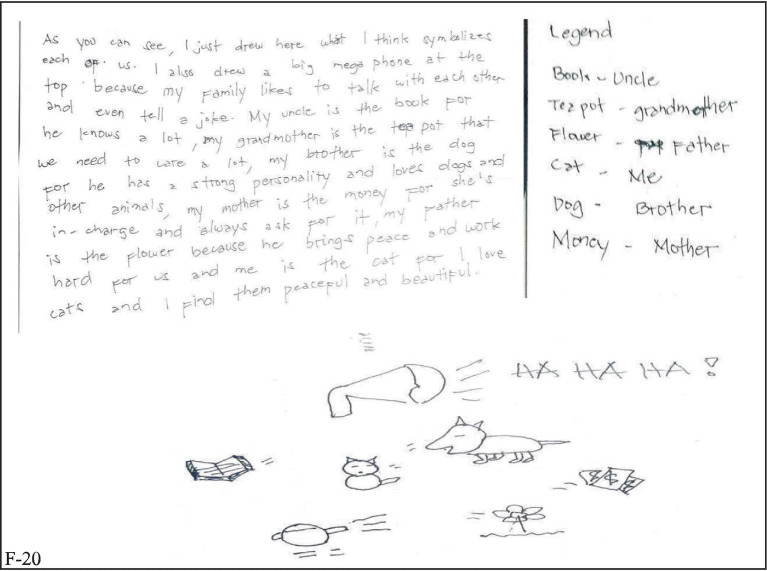
Open communication.

Having an extended family is a common cultural set-up in the country. For the respondents of this study, it contributed to their strengths to have open communication not only with their parents but with their grandparents, aunts, uncles, and cousins, as obvious from Figure 4. Open communication impacts social maladaptation (62), thus sharing thoughts, especially with family members, enhances the coping ability of a child toward varied external stressors.

**Figure 5:**
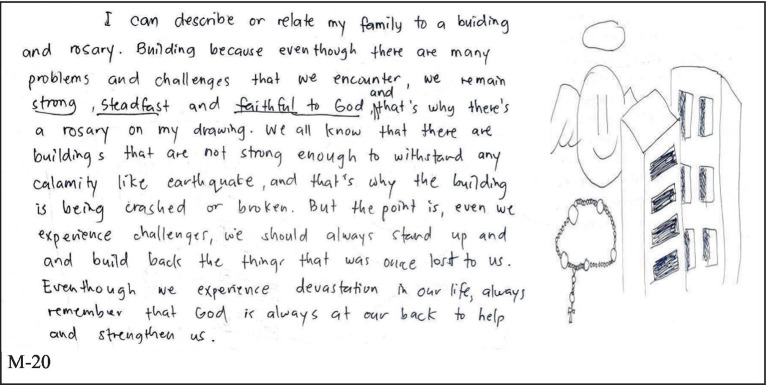
Faith/spirituality/belief in God or greater being.

Significantly, in Figure 5, respondents also mention faith in God or a Greater Being/spiritual belief as another factor that helps in their coping as they face daily challenges in life. Studies on faith-based intervention and mental health have found a positive correlation between the two variables (63–65). Therefore, considering the freedom of religion that is being enjoyed by citizens in this country, it may be helpful to consider spiritual disciplines in the implementation of mental health initiatives in academic institutions such as Bicol University.

**Figure 6:**
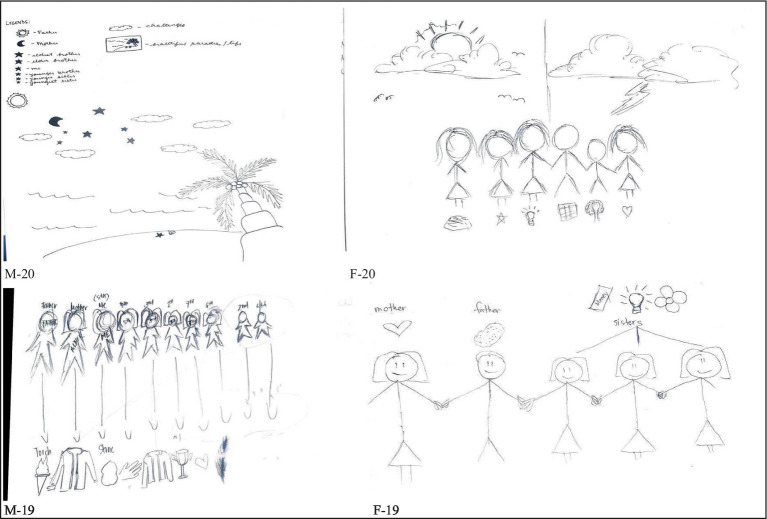
Familial bond.

Figure 6 also speaks of the dominant factor that influences the area of personal strength of the respondents—familial bond. The positive treatment of each other in the family is seen by the pre-service teachers as a significant step for them to learn how to respect others, cooperate, and act accordingly outside their homes. The illustrations and narratives of the intimacy of family members toward each other bring positivity in the student-participants’ personal outlook in life.

#### Vulnerabilities

**Figure 7:**
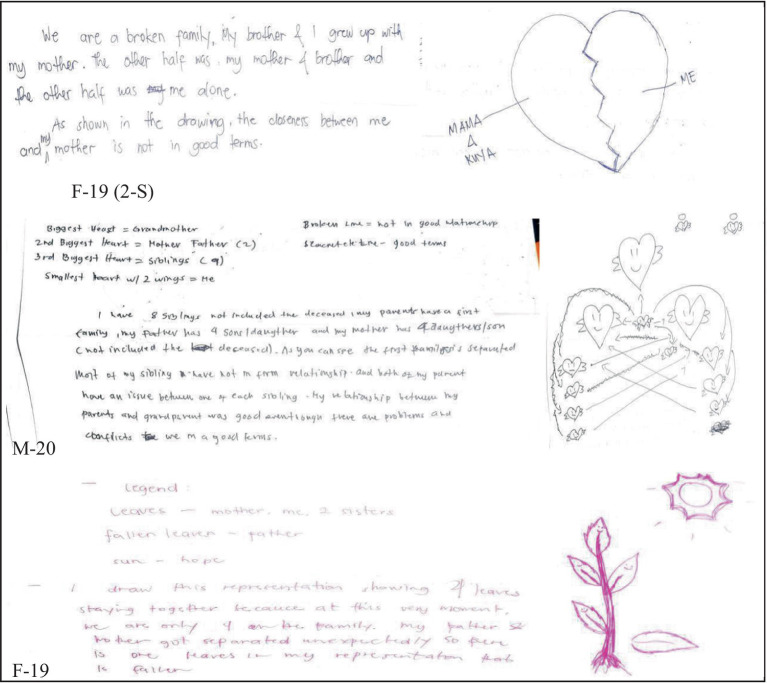
Broken family/parent’s separation (trust issues and betrayal).

In terms of factors that influence the area of personal vulnerabilities, the respondents of this study identified the separation of parents as the main contributor. A wealth of research has documented that parental divorce/separation is associated with an increased risk for child and adolescent adjustment problems, including academic difficulties (e.g., lower grades and school dropout), disruptive behaviors (e.g., conduct and substance use problems), and depressed mood (66, 67). Figure 7 supports previous literature as the pre-service teachers openly shared the pain and brokenness they felt due to their parents’ separation and the difficulty of coping up with the situation.

**Figure 8:**
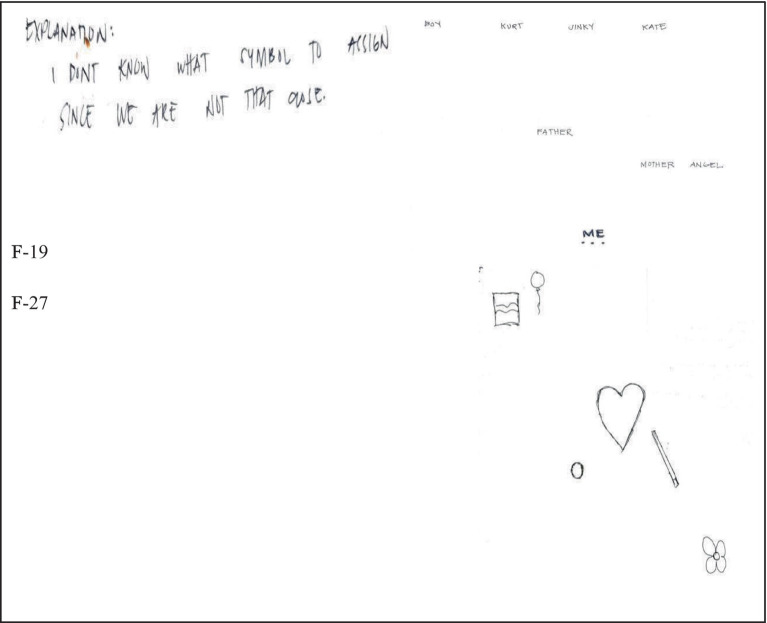
Psychoemotional distance.

Another factor disclosed by the participants is psycho-emotional distance. Students felt that they were not actually close with their parents or siblings because they do not communicate openly and frequently (Figure 8).

### Identify possible interventions suitable for their needs

Based on the insights given by the students during the FGD, the following interventions are deemed suitable for the needs of technical-vocational pre-service teachers: (1) A comprehensive assessment of the mental health status of the students. It will be necessary to explore or even create a relevant and contextualized instrument based on the needs of the present generation that will fully measure the mental health of technical-vocational pre-service teachers; (2) A consistent program/activity that will strengthen open communication among the students and with their teachers. The institutions may opt to adapt or adopt proven global initiatives on mental health for pre-service teachers; (3) A dialogue among students, parents, and faculty and staff to build strong psychoemotional connection that will support the holistic wellbeing of pre-service teachers. This could be a program of the guidance office to call for a quarterly special gathering that will just provide an avenue for students, teachers, and parents to communicate openly with one another; (4) A research center for students’ holistic wellbeing could be explored; (5) Mental health hotline, peer group support, and feedback wall (offline and online platforms) via the Guidance Center could be implemented; (6) Australia’s R U OK? Day could be adapted with permission and be celebrated during the mental health month in the Philippines or have our own Celebrate Wellness Program, Mental Health Day, and HAY (How Are You?) Day; (7) Craft mental health modules and workshops to be distributed face to face and online to students and teachers; (8) Administrators and teachers should consider the scheduling of subjects and deadline of tasks, respectively, considering the factors that may influence the performance of each learner; and (9) Faculty members may reevaluate activities to provide meaning and authentic learning experiences not just tons of tasks.

## Conclusion

The findings of the study were in line with the main objective of the research, which is to identify the psychological profile of BTVTEd pre-service teachers, identify their extreme life experiences, and present possible interventions and policy recommendations. The psychological profile of technical-vocational pre-service teachers reveals the dominant personality variable reported as areas of weaknesses—thinking disorder, persecutory ideas, deviation, and self-depreciation. At the same time, it also reveals the identified areas of strengths—cooperativeness, capacity for self-criticism, cheerfulness, and socially responsible attitudes. Familial relationships/household treatment and previous experiences at home and in school were found to contribute in the strengths and weaknesses of the respondents. Specifically, the factors that may have contributed to the students’ strengths are familial bonds or close family ties, open communication, and faith or belief in God or a Greater Being. Parents’ separation, which leads to broken family, trust issues, and the feeling of psychoemotional distance, influenced the personal area of vulnerabilities as reported by the respondents.

The culture of nurturing mental wellbeing and the openness to various practices and activities that help openly share one’s thoughts and authentic personality is still being developed in the college under study. Thus, further research focusing on school climate that impacts mental health should be taken into consideration. A comprehensive study on the demographic, relational, and peer factors that influence mental wellbeing should also be conducted for pre- and in-service teachers in the technical and vocational field in the country. Also, future research on the same variables could explore inferential statistics on psychological profile and gender, age, number of children in the family, and parents’ marital status and relationship. In addition, a comprehensive narrative and discourse analysis of textual data gathered in this study could also be explored in the future.

Finally, the following policy recommendations to promote mental healthcare, wellbeing, and resilience are highly encouraged: (1) Strengthen the application of mental health-related policies in the institution; (2) establish mental health care and holistic wellbeing centers across the institution; (3) support and fully funded student- and faculty-led mental health activities by crafting board resolutions and administrative orders; and (4) institutionalize support for Registered Guidance Counselors (RGCs), psychologists, and even psychiatrist (include in the annual budget so they can focus on research and programs about mental wellbeing).

### Strengths and limitations of the study

This study did not consider the general elements of mental health such as universal values. It only focused on the profiles or demographic backgrounds of second-year-major technical-vocational pre-service teachers, the fundamental personality variables incorporating basic cognitive and social skills and the ability to cope with adverse life events, and the relationship and feelings involved between the respondents and their family members. However, using a professionally accepted instrument (BPI) and mixed methods and textual analysis helped in the full exploration of the study and in the achievement of a well-documented representation of the mental health of the participants. Data collected through the administration of questionnaire were robust enough to explain some aspects of the complex issue of mental health in the institution under study.

Furthermore, the study is limited to 91 second-year students aged 18–35 years attending a state university in one of the regions in the Philippines and in using the BPI as the main measure. Since the study was restricted to one institution out of the 1,643 higher education institutions (HEIs) (68) in the Philippines, the results cannot be generalized to HEIs in the Philippines or overseas. Thus, this research, aimed for the relatability of the findings to other institutions in the country and the creation of institutional programs and policies that will cater to the mental health needs of students in the university.

Another limitation of this study was the use of a self-report instrument. Björkqvist et al. (69) stated that using self-reports in certain forms of personality variables such as thinking disorder might be under-reported because it is often unrecognized by the respondent and it is socially undesirable. Measures of social desirability in some cases lead to an assessment of bias in responding to the questionnaires (70, 72). On the other hand, as suggested by Chan (71), there is “no data that self-report data are inherently flawed and that their use will always impede our ability to meaningfully interpret correlations or other parameter estimates obtained from the data” (p. 330). For this study on the psychological profile of technical-vocational pre-service teachers, self-report is deemed appropriate since utmost consideration was given to ethical concerns to gain valid reports from the respondents. Memory error (70) was also considered as a limiting factor since the research asked the students to recall what happened to highlight their extreme life experiences.

Despite the identified limitations of this study, the highlight of this research is the actual data provided by the respondents themselves that served as the basis for the possible intervention and policy recommendations. Finally, this study could be a foundational source for future related research on mental health of pre- and in-service teachers primarily those with technical and vocational majors.

## Data Availability

The raw data supporting the conclusions of this article will be made available by the authors, without undue reservation.
